# Estimating trunk fat in children according to sex using basic somatic readings: an opportunity for improving evaluation among girls

**DOI:** 10.1186/s12887-021-02918-3

**Published:** 2021-10-11

**Authors:** Manuel Moya, Virginia Pérez-Fernandez

**Affiliations:** 1 Universidad Miguel Hernández, Health Sciences Campus of S. Juan, UMH Campus de S. Juan, Edificio Balmis, room S01 P002; Av Ramón y Cajal s/n. 03550 San Juan, Alicante, Spain; 2 Department of Surgery, Pediatrics and Obs & Gynecology, Facultad de Medicina, Universidad de Murcia, LAIB Building, Av. Buenavista s/n 30120 El Palmar, Murcia, Spain

**Keywords:** Abdominal fat, Easy anthropometry, Pediatric obesity, Sex distribution

## Abstract

**Background:**

The fat mass estimators waist-to-height ratio (WHtR) and relative fat mass—pediatric (RFMp) complement the widely accepted body mass index (BMI) in obesity evaluation. Aims of the Study: Conduct an easy appraisal of trunk fat and the cardiometabolic risk associated with pediatric obesity.

**Methods:**

A total of 472 children (39% boys in the total sample) were classified as underweight, normal weight, overweight or obese (nutritional groups, NGs) according to BMI Z-score after initial anthropometric data were obtained and ad hoc exclusion criteria were applied. WHtR and RFMp (% of total fat) were calculated for each group, associations were assessed through multiple linear regression (MLR), and differences between sexes were evaluated (medians, IQR).

**Results:**

The mean age (mean (95% CI)) was 10.8 y (10.1–11.1). The values in the total sample were as follows: WHtR, 0.5 (0.49–0.51) and RFMp%, 32.3 (31.7–33.0). In the overweight group, the values were as follows: WHtR, 0.51 (0.50–0.52) and RFMp(%), 34.2 (33.3–35.1). In the obese group, the values were as follows: WHtR, 0.56 (0.55–0.57) and RFMp(%), 37.8 (36.9–38.6). The associations were as follows (NG; independent variables): In the NG, adjusted R^2^ values were between 0.74 and 0.78. In the total sample, the beta coefficient was 3.36 (*P* < 0.001) for RFMp for girls; for waist circumference (WC), the beta coefficient was 2.97 (P < 0.001), and for WHtR the beta coefficients were − 0.01 (*p* < 0.001) and 0.03 (*p* < 0.001),for girls and for WC respectively.

The sex differences were as follows: BMI exhibited no differences in the NG (Mann-Whitney U). WHtR (median (IQR)) differed (M vs. F) in the total sample (0.49 (0.45–0.54) vs. 0.52 (0.45–0.56), *p* < 0.004); in the overweight group (0.51 (0.48–0.53) vs. 0.54 (0.51–0.55), *p* < 0.001); and in the obese group (0.55 (0.52–0.57) vs. 0.57 (0.54–0.60), p < 0.004). RFMp (%) differed in the total group (29.21 (24.27–32.92) vs. 36.63 (30.2–39.51), *p* < 0.001); in the overweight group (31.24 (28.35–32.35) vs. 37.95 (35.75–38.82), p < 0.001) and in the obese group (35.89 (32.05–36.15) vs. 40.63 (38.27–42.42), p < 0.001).

**Conclusions:**

WHtR and RFMp are simple and reliable indices that do not require centile charts. Their values, including waist circumference, can be used to estimate the different trunk fat components in boys and girls better than BMI, especially if individuals are overweight or obese. RFMp proved to be more reliable as it considers sex. Both should be included in routine anthropometric readings.

## Introduction

Excessive abdominal fat deposition is associated with obesity-related comorbidities in adults [1–3] and children and adolescents [4, 5]. Among children and adolescents, the most common consequences are elevated blood pressure and subtle metabolic disturbances, among the numerous and coexistent clinical deviations that may appear inconspicuously at these early moments. Body mass index, whether expressed as a Z-score or percentage (BMI Zs, BMI %), is a widely accepted measure to identify malnutrition in pediatric groups, but BMI cannot indicate present or future cardiometabolic risks in overweight or obese children; in contrast, in adults, waist circumference readings are favored [6]. Body composition is different between children and adults and is modified by obesity; assessing these changes is complex. T G Lohman in 1989 [7] described that these quantitative fat differences can be estimated by anthropometry, body density and bioimpedance methodology; regarding the latter [8], bioimpedance was used to estimate the fat-free mass in children and in adults according to sex and ethnicity. Moreover, through air displacement plethysmography [9], a young population (< 19 years) was studied and was also assessed on the same day with dual X-ray absorptiometry (DXA). Ten years later, [10] as DXA gained precision in the assessment of body composition, the use of some previous methods (underwater weight) and devices (Omron) was decreasing. Positron emission tomography-computed tomography (PET-CT), particularly magnetic resonance imaging (MRI), allows also the assessment of bone and muscle in studies [11]. Currently, in addition to the heritability of body composition [12], the previously described methods for studying body composition are still in use, but improved methodology has made a selection of them available for use in both research and clinical settings. Therefore, interest in using simple tests based on waist circumference that have shown a reasonable association with cardiovascular risks in children [13] and adolescents [14] and have been associated with the mentioned accurate methods of estimation of fat mass percentage has been increasing [15].

The prevalence of abdominal obesity is not very well known in pediatric groups because different methods (and names) are used to assessing abdominal obesity; the most common method is likely, dual X-ray absorptiometry (DXA) (central fat’ or ‘trunk fat’). Computed tomography and magnetic resonance imaging are considered the gold standard for quantitative measurement of abdominal adipose tissue compartments (visceral subcutaneous, etc.), although they are less frequently used due to minimal but significant radiation exposure and cost. Anthropometry is considered the basic and straightforward method, and pure waist circumference, apart from the far more commonly assessed BMI, is measured at each visit. Furthermore, abdominal fat increases as the child grows. After extensive research in adults [16, 17] evidencing an association of abdominal obesity with and/or predictive capacity for cardiometabolic conditions, waist circumference percentiles [18, 19] and diverse derived equations appeared in the pediatric obesity preventive literature. Of these, the waist-to-height ratio (WHtR) and relative fat mass—pediatric (RFMp) were selected for assessment in the present study. A WHtR [20] greater than 0.5 is associated with most health risks occurring in obese adults, even in subjects identified as normal weight. A value ≥0.5 has also been accepted for children and adolescents for the estimation of abdominal obesity. In the context of relative fat mass (RFM), obesity risks depend on an elevated ratio of adipose tissue mass to total body weight. Therefore, precise estimation of body fat percentage is relevant. RFM better predicts whole-body fat percentage measured by DXA in males and females. RFM was developed in a large study on adults [21] RFM—pediatric (RFMp) has been developed also [22]. RFMp is also an estimator of fat mass percentage based on the height/waist ratio with the presumed advantage of considering sexes separately and has exhibited close agreement with DXA measurements. Therefore, RFMp can quickly provide an idea of fat content not only at initial diagnosis but also over the course of long-term obesity follow-up.

Our hypothesis was as follows: because the correlations of trunk fat estimators with matched DXA data have been established, we hypothesize that WHtR and RFMp are elevated in pediatric overweight and obese individuals, thus signaling cardiometabolic risk in both sexes.

The aim of this study was to determine whether these clinically matched estimators can provide information about the (trunk) fat content in addition to the information provided by globally recognized BMI in children with different nutritional statuses (underweight, normal weight, overweight and obese), especially signaling fat difference thresholds by sex.

## Methods

### Study design

Secondary analysis of exclusively initial diagnostic measures of patients attending the Pediatric Institute for Nutrition, Growth and Metabolism Clinical Unit of the University Hospital was conducted. Participants: A total of 472 (185 boys) children and adolescents aged 10.8 years (95% CI 10.1–11.1) were classified into nutritional groups according to their BMI Z scores as underweight (UW, < − 1 SD), normal weight (NW, − 1 to + 1 SD), overweight (OW, + 1 to + 2 SD) and obese (OB, > 2 SD). Age and sex subgroups were also studied. These baseline data came from patients attending the unit between 2014 and 2018. All of the participants were followed and cared for under the direction of this unit. Children with incomplete somatic data, low school performance, atypical social status and chronic conditions were excluded (*n* = 27).

### Intervention

According to the established rules of the unit, the following anthropometric measures were taken by specialized and specific personnel: height (Harpenden stadiometer, Holtain Limited, Harpenden UK), weight (electronic scales), waist circumference (inextensible tape) and blood pressure (GE Carescape tm, V100, Dinamap Technology, Freiburg, Germany, two pressure cuffs). For waist circumference (WC), we followed the recommendations of the WHO [23] but with pediatric precautions as follows: the child was in a standing position, and the tape was horizontally placed at the midpoint between the lower costal margin and upper anterior iliac apophysis. The tape was not too tight or too loose and reading to the nearest 0.1 cm at the end of exhalation was obtained, but before recording the result, changes in the centimeter readings were assessed (left to right) with respiratory movements. All measures were taken in an acclimatized room where children were in light underwear and barefoot, always in the morning and after a light continental breakfast. For height and WC, all readings were in centimeters and centiles, and Z scores were obtained through the anthropometric program based on IOTF standards. BMI % was also assessed according to the Poskitt definition [24]. Target height ([25] Molineri 1984) was assessed as midparental height ± 6.5 cm for both boys and girls. WHtR is a unitless ratio. RFMp was calculated according to pediatric equations [26], and the results express the estimated percentage (%) of total body fat:

RFMp (for 8 to 14 years) = 74 – (22 x height/waist) + (5 x sex);

RFMp (for 15 to 19 years) = 64 – (20 x height/waist) + (12 x sex).

Note that for both equations, sex implies male 0 and female 1, and the results are given [26] as percentages. Because there is no definition of obesity based on body fat percentage, we used NHANES data matched to DXA as a reference (22), where figures greater than 29% for boys and 41% girls would indicate high body fat percentage. In our sample, values very close to the nutritional groups defining limits were obtained (Table 1), indicating the association of overweight with greater abdominal adiposity (trunk fat).

**Table 1:** Main clinical data (mean 95% CI) at baseline for all patients and for BMI nutritional groups

	ALL *n* = 472 (39% boys)	UNDER W *n* = 35 (45% boys)	NORMAL W *n* = 182 (39% boys)	OVER W *n* = 112 (41% boys)	OBESE *n* = 143 (58% boys)
**Birth Weight** (g)	3108.4 3062.2–3154.6	3061.4 2943.5–3178,5	3186.3 3119.4–3253,2	3117.1 3012.0–3222.2	3010.6 2922.9. - 30,984
**Birth Weight** (Z score)	− 0.04 − 0.13–0.05	0.0006 − 0.25–0.25	0.08 − 0.06–0.22	− 0.11 − 0,31–0.09	0.17 − 0.35–0.01
**Age** (years)	10.8 10.5–11.1	11.9 10.7–12.3	11.3 10.8–11.7	10.3 9.9–10.7	10 .3 9.8–10.8
**Systolic BP** (mmHg)	108.8 107.5–110.1	104.5 101.4–107.6	108.2 106.5–109.9	108.8 105.5–110,9	110.5 107.2–113.8
**Diastolic BP** (mmHg)	63.8 62.7–64.9	58.9 53.7–62.9	63.2 61.7–64.6	63.8 61.7–65.9	65.6 63.3–67.2
**Waist C** (Centile)	72,9 70.2–75.6	24.7 18.3–31.3	56.6 52.5–60.7	86.4 83.6–89.2	94.8 92.7–96.7
**Waist C** (Z score)	1.2 1.06–1.35	− 0.81 − 1.03–0.6	0,29 0,15–0.43	1.49 1.3–1.7	2.6 2.4–2.8
**WHtR**	0.5 0.49–0.51	0.41 0.40–0.41	0.46 0.45–0.47	0.51 0.50–0.52	0.56 0.55–0.57
**RFMp** %	32.3 31.7–33.0	22.8 21.2–24.4	28.9 28.1–29.7	34.2 33.3–35.1	37.8 36.9–38.6
**BMI%**	112.1 110.5–113.7	80.6 79.6–81.7	99,6 98.6–100.6	114.5 113.9–115.0	134.1 131.9–136.3
**BMI** (Z score)	0.91 0.78–1.02	− 1.35 − 1.46- -1.24	− 0.02 − 0.05–0.09	1.05 1.0–1.2	2.5 2.3–2.7

Abbreviations: *W* refers to weight; *WHtR* Waist-to-Height Ratio; *RFMp* Relative Fat Mass pediatric; *BMI %* BMI percentage

BMI Z scores were selected as a general and widely accepted model but did not include waist circumference, whereas WHtR and RFMp provided an idea of trunk fat.

### Ethics

The applied procedures were conducted in accordance with the standards of the institutional Ethics Committee and with the Declaration of Helsinki (1964; 2000). The study was approved by our Institution Ethics Committee.

### Statistical analysis

A descriptive analysis was performed, and the mean and standard deviation or 95% confidence interval or median and interquartile ranges (IQRs) are provided for quantitative variables according to the previous results of a normality test. For categorical data, absolute values and percentages were applied. A univariate analysis by the Mann–Whitney U test to assess sex differences in estimated fat percentages was also applied to dependent and independent variables, as shown below. Correlation studies were conducted to assess the linear relationship among the dependent and continuous covariates. A multivariate study was conducted to assess the relationships of covariates (sex, BMI Zs, waist circumference (WC Zs), birth weight (BW Zs), systolic and diastolic blood pressure) with dependent variables (WHtR and RFMp). Next, the B (Beta) coefficient was obtained for each dependent variable in five models, four according to every BMI group and the fifth comprising all participants without any stratification. Stata Biostatistical Program, SSS version 15, 2017, was used, and a *P* value < 0.05 was considered significant. The findings of this study should be considered exploratory and/or descriptive.

## Results

Table 1 shows the values for the main clinical data of the total sample and the following nutritional status groups: underweight, normal weight, overweight and obese. The total sample was considered because of the potential association of estimators with a wider range of BMIs. All nutritional groups included both sexes, and their clinical values are shown according to BMI nutritional categories. It is worth noting the narrow 95% confidence interval for all values. RFMp varied from 22.3% in the underweight group to 37.8% in the obese group, and WHtR and waist circumference Z scores also exhibited narrow ranges in agreement to their nutritional status.

Because of the higher values of body fat percentage in females, Table 2 shows the different degrees of association of sex with fat estimators in the analyzed groups. As expected, no sex difference appeared for the BMI Z value, as it was used as a primary categorizer for underweight, overweight and obesity among the total sample of children with a mean age of 10.8 years (95% CI 10.5–11.1), but these Z score values (Table 1) provide baseline data related to fat mass differences. WC Z scores were higher and exhibited significant levels in girls in all groups, even in the underweight group (*p* < 0.004). Concerning (trunk) fat mass estimators, WHtR was greater in girls than in boys in the total sample (p < 0.004), specifically in the overweight (*p* < 0.001) and obese groups (p < 0.004). RFMp showed differences in the total sample and the normal weight, overweight and obese groups, with higher values in females (p < 0.001). All these sex differences were observed with no differences in BMI within the groups, suggesting it is worth considering that fat plays a role in body weight in addition to other components.

**Table 2:** Sex differences in fat mass estimators in nutritional groups

	ALL	Under Weight	NORMAL WEIGHT	OVER WEIGHT	OBESE
N (% BOYS)	472 (39.4)	35 (45.7)	182 (39.0)	112 (41.1)	143 (37)
**BMI Zs (a)**					
M	0.86 (− 0.23/1.63)	− 1.42 (− 1.78/−.24)	− 0.04 (− 0.57/0.37)	1.09 (0.91/1.27)	2.23 (1.67/2.97)
F	0.81 (0.12/1.67)	− 1.28 (− 1.47/− 1.15)	0.14 (− 0.31/0.39)	1.02 (0.84/1.20)	2.33 (1.73/3.36)
U	2135	93	3201	1291	2135
p value	0.444	0.135	0.079	0.245	0.444
**WC Zs (a)**					
M	0.59 (− 0.32/1.51)	− 1.21 (− 1.49/− 0.93)	− 0.22 (− 0.74/0.31)	0.88 (0.30/1.33)	1.80 (1.35/2.44)
F	1.73 (0.38/2.67)	− 0.33 (− 1.22/0.02)	0.40 (− 0.24/1.40)	2.03 (1.42/2.50)	3.20 (2.34/3.20)
U	16,429	66	2412	441	960
P value	0.001	0.004	0.001	0.001	0.001
**WHtR (a)**					
M	0.49 (0.45/0.54)	0.41 (0.40/0.42)	0.46 (0.43/0.48)	0.51 (0.48/0.53)	0.55 (0.52/0.57)
F	0.52 (0.45/0.56)	0.41 (0.40/0.43)	0.46 (0.43/0.51)	0.54 (0.51/0.55)	0.57 (0.54/0.60)
U	1693	3449	3449	931	1692
P value	0.004	0.155	0.115	0.001	0.004
**RFMp%**(a)					
M	29,21 (24.27/32.92)	20.73 (18.67/21.37)	25.69 (22.80/28.20)	31.24 (28.35/32.35)	35.89 (32.05/36.15)
F	36.63 (30.2/39.51)	25.05 (23.44/28.39)	30.52 (28.20/35.09)	37.95 (35.75/38.82)	40.63 (38.27/42.42)
U	1803	1412	931	142	372
P value	0.001	0.001	0.001	0.001	0.001

Abbreviations: *a*, median (IQR); *U* Mann–Whitney U; *M* male; *F* female; *WC* waist circumference; *Zs* Z-score

The associations of trunk fat estimators (WHtR and RFMp) with the groups and with the main independent variables in these groups are shown in Table 3. This was evaluated in two ways. First, the adjusted coefficient of determination (aR^2^, table first column in both estimators) was considered. In the case of WHtR, the aR^2^ values explained its association with the BMIs that characterize each group, ranging from 0.88 in the total sample to 0.007 in the underweight group. When considering aR^2^ for RFMp, the association in all studied groups ranged from 0.87 in the total sample to 0.57 in the underweight group, signaling a certain advantage of this latter estimator; both estimators explained nearly 88% of the outcomes. Second, in the multivariate study (subsequent columns in Table 3), regarding the B coefficient, as clearly appears in the case of WHtR values, the associations with the six variables analyzed in each nutritional status group were weaker, indicating a weaker association with some variables, especially birth weight and systolic and diastolic blood pressure. As expected for RFMp, the B coefficient had high values for sex in the four nutritional groups and the total sample, ranging from 3.77 in the obese group to 4.58 in the underweight group and 3.36 in the total sample, signaling greater values for girls regardless of nutritional group to which they belonged, thus indicating a significant association with trunk fat.

**Table 3:** Association between trunk fat estimators and nutritional groups and independent variables in these groups

WHtR					RFMp				
Group	Indp Var	B Coef	95% CI	*P* value	Group	Indp Var	B Coef	95% CI	*P* value
**All** aR2 = 0.880	Sex	−0.016	− 0.021 - -0.010	0.001	**All** aR2 = 0.872	Sex	3.363	2.733–3.994	0.001
	BMI Zs	0.009	0.006–0.013	0.001		BMI Zs	0.765	0.378–1.152	0.001
	WC Zs	0.031	0.027–0.035	0.001		WC Zs	2.971	2.584–3.357	0.001
	Birth W Zs	−0.003	−0.006 - -0.001	0.001		Birth W Zs	−0.206	− 0.465–0.051	0.117
	Sys BP	0.00008	−0.00004–0.0002	0.211		Sys BP	0.009	−0.004–0.023	0.190
	Dias BP	0.00001	−0.0001–0.0001	0.804		Dias BP	−0.0002	−0.015–0.015	0.972
**Under W** aR2 = .007	Sex	−0.003	−0.020–0.013	0.66	**Under W** aR2 = .573	Sex	4.585	2.208–6.962	0.001
	BMI Zs	0.018	−0.012–0.048	0.22		BMI Zs	2.055	- 2.143 – 6.254	0.315
	WC Zs	0.007	0.007–0.022	0.278		WC Zs	1.211	−0.825–3.268	0.224
	Birth W Zs	−0.003	−0.011–0.004	0.407		BW Zs	−0.450	- 0.153–0.632	0.391
	Sys BP	< 0.001	0.0004–0.0007	0.603		Sys BP	0.013	−0.064–0.090	0.723
	Dias BP	< 0.001	−0.0003–0.0007	0.503		Dias BP	0.036	−0.037–0.109	0.315
**Normal W** aR2 = 0.657	Sex	−0.017	−0.026–0.008	0.000	**Normal W** aR2 = .740	Sex	2.997	2.040–3.953	0.001
	BMI Zs	0.0004	−0.005–0.015	0.307		BMI Zs	0.919	−0.255 – 2.096	0.124
	WC Sz	0.034	0.027–0.041	0.000		WC Zs	3.460	2.717–4.203	0.001
	BirthW Zs	0.003	−0.007–0.0007	0.112		Birth W Zs	−0.265	−0.697–0.166	0.226
	Sys BP	0.00008	−0.00001–0.0008	0.472		Sys BP	0.013	−0.010–0.037	0.266
	DiasBP	0.0001	−0.00004–0.0003	0.217		Dias BP	0.0009	−0.016–0.036	0.470
**Over W** aR2 = 0.606	Sex	−0.013	− 0.023 - -0.0008	0.064	**Over W** aR2 = .823	Sex	3.602	2.392–4.812	0.001
	BMI Zs	0.014	−0.004–0.032	0.128		BMI Zs	1.107	−0.439 – 2.654	0.158
	WC Zs	0.032	0.025–0.040	0.001		WC ZS	2.918	2.290–3.547	0.001
	Birth W Zs	−0.007	−0.012 - -0.002	0.007		Birth W Zs	−0.710	−1.154 - -0.266	0.002
	Sys BP	0.0001	0.0001–0.0004	0.333		Sys BP	0.009	−0.014–0.033	0.452
	Dias BP	0.00049	−0.0004 - 0.0002	0.573		Dias BP	−0.004	−0.031–0.022	0.744
**Obese** aR2 = 0.760	Sex	−0.015	−0.026 - -0.004	0.008	**Obese** aR2 = 0.752	Sex	3.767	2.588–4.947	0.000
	BMI Zs	0.008	0.002–0.014	0.006		BMI Zs	0.512	−0.108 – 1.133	0.105
	WC Zs	0.028	0.021–0.034	0.001		WC ZS	2.281	1.619–2.943	0.001
	Birth W Zs	−0.001	−0.005–0.002	0.390		Birth W Zs	0.293	−1.138–0.725	0.181
	Sys BP	5.1e-06	−0.0002 - 0.0002	0.965		Sys BP	0.002	−0.021–0.026	0.866
	Dias BP	−5.1e-06	−0.0003–0.0001	0.632		Dias BP	−0.016	−0.041–0.009	0.219

Abbreviations: *W* refers to weight; *aR*^*2*^ coefficient of determination R squared; *B coef* indicates the expected amount of change for every unit increase in each independent variable

Standard correlation matrix: In the normal weight group, BMI was correlated with trunk fat estimators; the strongest association was found for WHtR (r = 0.63; *p* < 0.001) and RFMp (r = 0.58, *P* < 0.001). These trends were maintained in the overweight and obese groups. The correlation matrix values did not show any further remarkable findings.

## Discussion

The main findings of this study are as follows: 1) Waist circumference (WC), waist-to-height ratio (WHtR) and relative fat mass—pediatric (RFMp) can be used to estimate truncal fat because the models used indicate that their change is associated with the six analyzed independent variables (Table 3); this association was moderately stronger in the total sample, consequently adding valuable information to the BMI estimative capacity of body fat content. 2) The estimators exhibited differences between boys and girls in all nutritional groups, whereas BMI did not exhibit differences. The nonstatistical significance, in the case of BMI according to one of the statistical principles [27], cannot be interpreted as equality, as this was a primary idea in this study. 3) Mean values and uncertainty ranges of estimators were obtained in each nutritional group.

The risk associated with excessive trunk fat was described in the mid-1900s in adults [28], stressing the importance of the body shape of the individuals. These risks were shown later in the context of a wide range of comorbidities. Paradigmatic examples are the associations with type 2 diabetes [29, 30], hyperuricemia [31], elevation of free T_3_ and MRI-assessed abdominal fat distribution [32], heart failure mid-range ejection fraction [33], and even prevalent or previous metabolic syndrome. These relationships were established by means of different waist circumference-derived indices [34]. It is worth referring to the conclusions of Baton Rouge [35] regarding the analysis of these various equations; although the waist circumference index is preferred, capacity of indices for evaluating an individual person’s health risks were considered. To improve feasibility, other waist-height indices may be useful [36] and have already been tested in different geographic areas in children as cardiometabolic risk factors [37]. More specifically and due to the simplicity and reliability of measures, WHtR was chosen for assessing central adiposity in children in a remote South Pacific archipelago [38]. With the present-day understanding of pediatric obesity risks, it is worth considering that these elevated trunk fat markers are associated with the main biochemical markers of insulin resistance and inflammatory and metabolic abnormalities [39]. The clinical approach of analyzing trunk fat has led to the assessment of 17,000 participants with BMI < 25 kg/m^2^ but with excessive body fat [40]. In children and young people, the estimation of trunk fat by proxy methods has been slow due to the varied charts for waist circumference despite the publications of McCarthy [41, 42] facilitating Z score calculation. More recent publications [18, 43] included international centile cutoffs, but nevertheless, truncal assessment has not reached the accepted level of BMI in a clinical setting. Waist circumference is still considered a reliable measure for assessing abdominal obesity [44], especially in countries with uneven care distribution, and in others with better conditions, its evaluation is the first or preliminary step prior to subsequent more precise tests [45].

### Waist-to-height ratio

WHtR in adults was proposed in 1995 in Japan by SD Hsieh and almost simultaneously in the UK by M Ashwell [20], demonstrating that ratios > 0.5 were strongly associated with myocardial ischemia and metabolic risk factors (T2D). This association has also been described in children and adolescents elsewhere [46, 47]. Other variants of this ratio [48] are not widely used. In adolescents in the AVON longitudinal study [14] that included nearly 3000 children followed over 8 years, ratios > 0.5 were associated with elevated fasting blood lipids, glucose, insulin and blood pressure in boys (OR 6.8; 95% CI 4.4–10.6) and girls (OR 3.8; 95% CI 2.3–6.3), and the associations of this ratio once established were highly specific compared to those of BMI. Similar results were shown in a systematic review and meta-analysis [49]. Consequently, WHtR could be considered a simple and reliable first step in risk assessment.

### Relative fat mass—pediatric (RFMp)

**Fig. 1:**
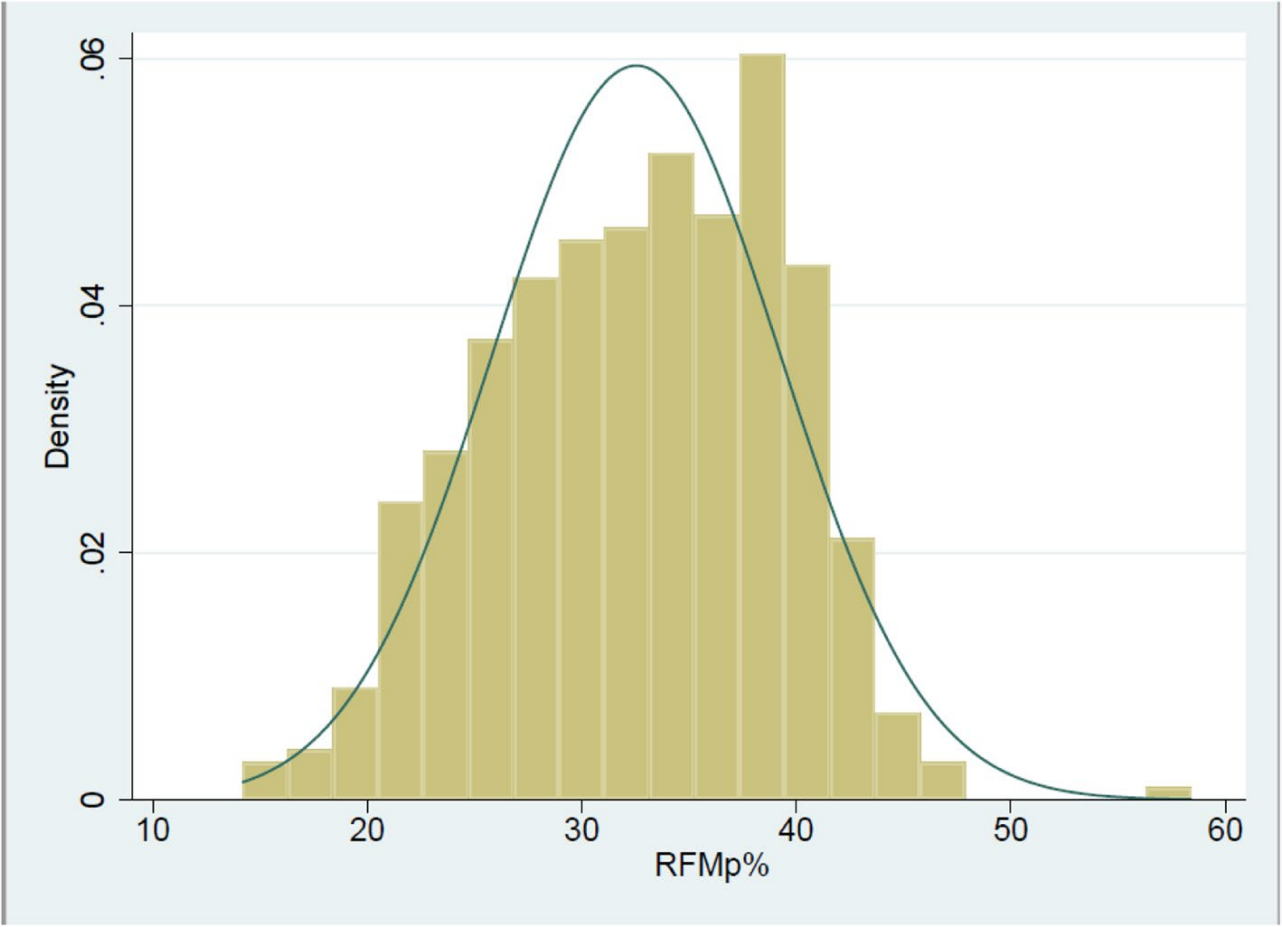
Relative Fat Mass–pediatric (RFMp %). Normal distribution histogram in the whole sample. The density curve is related to the probability of upcoming random variables

As mentioned, Woolcott and Bergman [21] derived an equation from adult height/waist for estimation of whole-body fat percentage and later developed an equation for children and adolescents [22], which was assessed according to DXA values. The novelty of this estimator is the sex consideration, which decreases the rate of misclassification of relative fat mass due to a more precise diagnosis of obesity/adiposity in females. This equation has been tested in other parts of the world [50, 51] in adult populations and in adolescents [52]. In our study, the initial correlation with BMI as the major standard criterion for overweight and obesity classification was significant in the whole sample and normal weight groups, but in the overweight and obese groups, the degree of correlation slightly decreased, which was in agreement with the next multiple regression finding. This fact is interesting because waist circumference does not intervene in the measurement of BMI and would be more related to body or trunk fat than BMI. The normal distribution and density of RFMp in this study could provide adequate conditions for future analyses (Fig. 1).

### Multiple linear regression

The high aR^2^ values are indicative of the appropriateness of the estimators used, but since they are below 90% (predictive capacity), they should be considered indicative of association mainly for females and waist circumference and, to a lesser extent, BMI Z scores. Specific analyses of nutritional statuses revealed that in the total sample, among individuals with greater fat deposit (obese) and the lowest fat deposit (underweight), an association between sex and RFMp was found; the regression B coefficients implied that girls have an RFMp 3.36 units higher than boys, or in the case of WC Z scores, each unit of increment implies an increase of 2.97 in the RFMp. This associative trend was very similar and regular in the normal weight, overweight and obese groups. All of these findings indicate greater precision than that of BMI Z score, basically because BMI does not consider waist circumference, which is also manifested through its lower coefficients (Table 3). For WHtR, these associations remain at a lower level but maintained their *p* values; therefore, the simplicity of its calculation (ratio waist/height) and its well-proven threshold of 0.5 make it an efficacious screening tool.

In the underweight group (7.4% of the total sample), all children were discreetly affected (BMI Zs − 1.35 SD; 95% CI − 1.46 to − 1.24), with a minor reduction in target height (− 0.05 SD; 95% CI − 0.23 to 0.25) in 20 instances where both progenitors were measured, suggesting potential familial undernutrition; furthermore, their social level could not be considered as of lower class. Inclusion in the study was motivated to assess the estimators’ behavior on the opposite side of the spectrum of overweight.

Regarding sex, in all nutritional groups, BMI did not show differences between sexes; conversely, the estimators clearly did, as weight apart from fat comprises nonfat body components that veil adiposity. This would justify the increasing values of RFMp in the normal, overweight and obese groups; again, this no difference occurs with BMI Z scores in both boys and girls. The median RFMp was significantly higher in girls than in boys, sharing the same classificatory BMI range in all groups (regardless of the BMI category), which is probably in agreement with the higher fat content in girls at this age [22], consequently giving an idea of abdominal fat.

Furthermore, DXA studies revealed a greater proportion of fat in girls, particularly when they reach puberty [53, 54]. This association with DXA was already studied by us [55] in 142 overweight and obese individuals with an average age (mean, 95% CI) of 11.5 (10.3–11.8) years, with no differences between boys and girls in age or BMI Z score; furthermore, in that study, we found a relationship between %trunk fat and sex—42.2% (40.3–44.1) in boys versus 45.8% (43.7–47.8) in girls (*p* = 0.001)—and for waist circumference (WC) Z score—1.9 (1.7–2.2) in boys versus 2.4 (2.1–2.8) in girls (p = 0.001); the regression analysis between WC and %trunk fat revealed a regression coefficient of β = 2.9 for WC (*P* = 0.001). Thus, there is a need to study sexes separately in pediatric obesity studies.

### Blood pressure

Only a weak relationship of diastolic blood pressure with RFMp (r = 0 .206, *p* < 0.001) appeared in the total sample. Despite well-established policies for BP measurement in the clinical area of this unit with an elevated blood pressure section [56], the results are not as consistent as those of other clinical parameters, and doubts about BP screening [57, 58] probably not only apply to these data but also lead to reconsidering these policies.

Present techniques allow abdominal fat to be measured separately from subcutaneous fat in children [59, 60], but this may not be an available method for evaluation of the growing double burden of malnutrition in low- and middle-income countries (LMICs), so it is important to look for proxy estimators. Furthermore, proxy measures are useful even when DXA is available but cannot be justified at every follow-up visit. Therefore, and after firm association of estimators based on waist circumference and trunk fat, we recommend that the studied estimators be used because of their safety, simplicity [61], known low variability [62] and good correlation with CT and MRI [63] when assessing metabolically unhealthy fat accumulation [3, 64] and furthermore to increase precision by considering sex [55] and age if assessing a diverse sample [65, 66, 67].

### Limitations

Using accurate standardized anthropometric data, we assessed the potential advantages of WHtR and RFMp without a specific design for child obesity studies [68]. As females were predominant (286/472, 60.6%), the subsequent higher proportion in the study groups could be a confounding factor, and it was not possible to perform validation [69]. Therefore, another weak point is the lack of a precise comparison, such as DXA, but in this case, the aim was to assess diagnostic performance of anthropometry for nutritional deviations, some of them not requiring more complex techniques with side effects.

## Conclusions

The calculation of WHtR is a very simple and reliable method that does not require reference growth centile charts; consequently, it should be the first step in screening, while RFMp gives an idea of the body (and trunk) fat content in both sexes. Both could indicate cardiometabolic consequences that are already present or that could occur in the near future, especially if the values increase during the follow-up. At present, BMI Z score is considered the most widely used marker for overweight and obesity (while BMI percentage is better understood by patients and their families); hence, to increase clinical accuracy, both estimators should be added as routine anthropometric measurements in primary health care settings and in specific surveys.

## Data Availability

The generated dataset is available from the corresponding author on reasonable request.

## Abbreviations

BMI%: Relative body mass index
MLR: Multiple linear regression
NGs: Nutritional groups
OB: Obese
OW: Overweight
RFMp: Relative fat mass index—pediatric
WC: Waist circumference
WHtR: Waist-to-height ratio
